# Complete genome assembly data of *paenibacillus* sp. RUD330, a hypothetical symbiont of *euglena gracilis*

**DOI:** 10.1016/j.dib.2020.106070

**Published:** 2020-07-25

**Authors:** Victoria Yu. Shtratnikova, Yulia A. Rudenskaya, Evgeny S. Gerasimov, Mikhail I. Schelkunov, Maria D. Logacheva, Alexander A. Kolesnikov

**Affiliations:** a Belozersky Institute of Physico-Chemical Biology, Lomonosov Moscow State University, Leninskie gory, b.1, h. 40, Moscow, 119991, Russian Federation; b Biological faculty, Lomonosov Moscow State University, Leninskie gory, b.1, h. 12, Moscow, 119991, Russian Federation; c Skolkovo Institute of Science and Technology, Nobel St. 3, Moscow 143026, Russian Federation; d Institute for Information Transmission Problems, Russian Academy of Sciences, Bolshoy Karetny per., h. 19, b. 1, Moscow, 127051, Russian Federation; e Martsinovsky Institute of Medical Parasitology, Tropical and Vector Borne Diseases, Sechenov University, Trubetskaya str., h. 8, b.2, Moscow, 119991, Russian Federation

**Keywords:** *Paenibacillus*, Illumina, Nanopore, NGS sequencing, Genome assembly

## Abstract

An unknown bacterial strain was detected in the cytostome of *Euglena gracilis* and on the cell surface of *Euglena gracilis* using transmission electron microscopy. To identify the unknown bacterium and its function, we performed isolation experiments. Here we present the genome sequence of the isolate that was determined to be *Paenibacillus* sp. The genome of the bacterium was sequenced four times using Illumina technology with pair-end reads, Illumina technology with mate pair reads (inserts 3–4 and 6–8 Kb), and Nanopore technology with long reads (tens of thousands of nucleotides). Assemblies based on Illumina reads including mate-pair reads could not resolve issues caused by long tandem copies of rRNA, other tandem repeats, and extremely GC-rich regions (90–100%). Only long Nanopore reads resolved those gaps and made it possible to complete the entire genome; moreover, we found one plasmid. The length of the genome is 5.56 Mbp, and the average GC content is 59%. The genome of *Paenibacillus* sp. RUD330 included 8 copies of all the rRNA genes (23S; 16S; 5S), the length of the plasmid was 8.3 Kb.

We hope that our genome assembly and the methods used can help other investigators in the assembly of complex genomes. Our reliable assembly could be a good basis for further physiological and genetic engineering studies of similar strains.

**Specifications Table**

**Table:** 

**Subject**	Microbiology
**Specific subject area**	Genomics of bacteria
**Type of data**	Table
	DNA sequence
**How data were acquired**	Instruments: Microbial cultivation, DNA isolation, NGS library preparation, Illumina HiSeq2000, Nanopore MinION flowcell R10; software packages *NextClip* v.0.8, *Trimmomatic* v.0.32, *Guppy* 3.4.3, *Velvet* v.1.2.08, *SPAdes* v.3.6.0 and 3.13.0, *CLC Genomics Workbench* v.8.5, *Newbler* v.2.9, *GapFiller* 1.10, *Unicycler* v.0.4.8, *BioEdit*.
**Data format**	Raw
	Analyzed
	Filtered
**Parameters for data collection**	The medium with *Euglena* culture was streaked on Petri dishes with selective medium. *Euglena* was eliminated, while the cohabiting bacterium showed resistance to antibiotics. Several colonies were picked and transferred to solid and liquid media. The bacteria were grown on liquid LB medium for NGS sequencing.
**Description of data collection**	DNA was isolated using the DIAtom DNAprep 100 kit (Izogen, Moscow). The sequencing library with an insert size of 300–400 bp was prepared using the TruSeq DNA sample preparation kit (Illumina, USA) after the ultrasonic fragmentation of genomic DNA with Covaris S220. Two mate pair libraries with insert size ranges of 3000–4000 and 6000–8000 bp were created with the Nextera mate pair sample preparation kit (Illumina). The libraries were sequenced on Illumina HiSeq 2000, generating paired-end reads of 100 nt.
	The library for Nanopore technology was prepared out of non-fragmented total genomic DNA using NEB Next Ultra II DNA library kit (NEB, UK) and Ligation Sequencing kit 1D (Oxford nanopore technologies, LSK-109), barcoded using Native barcoding kit, and sequenced on MinION, R10 flowcell (Oxford nanopore technologies).
	*De novo* genome assembly with Illumina and Nanopore reads was performed with the software described in “**How data were acquired”**
**Data source location**	Institution: Lomonosov Moscow State University, Department of Molecular Biology
	City/Town/Region: Moscow
	Country: Russian Federation
	Latitude and longitude (and GPS coordinates) 55.45 N 37.37 E
**Data accessibility**	Repository name: NCBI Genbank
	Data identification number: Genome and annotation: CP022655.2; Reads: PRJNA396653, including SRX5491169 (paired-end); SRX5491170 (mate pair with 3–4 kB insert size); SRX5491171 (mate pair with 6–8 kB insert size); SRR10950300 (Nanopore reads).
	Direct URL to data: Genome and annotation: https://www.ncbi.nlm.nih.gov/nuccore/CP022655.2; Reads: SRA, https://www.ncbi.nlm.nih.gov/sra/PRJNA396653, including https://www.ncbi.nlm.nih.gov/sra/SRX5491169 (paired-end); https://www.ncbi.nlm.nih.gov/sra/SRX5491170 (mate pair with 3–4 kB insert size); https://www.ncbi.nlm.nih.gov/sra/SRX5491171 (mate pair with 6–8 kB insert size); https://www.ncbi.nlm.nih.gov/sra/SRR10950300 (Nanopore reads).

**Value of the data**

• Our reliable assembly could be a good basis for physiological, phylogenetic, and genetic engineering studies. The description of the methods used can help in the assembly of complex genomes.
• The data provided in this article could be useful for microbiologists, genetics, genetic engineers, ecologists.
• The assembled genome can be used for the search of certain genes, transcriptional factors, transcriptomic investigations, and strain and species comparisons.
• We describe the challenges encountered in the assembly of this genome, and we hope that our solutions will help researchers facing the same problems.

## 1 Data description

A bacterial strain was detected in the cytostome of *Euglena gracilis* and on the cell surface of *Euglena gracilis* using transmission electron microscopy. The environmental interactions between *E. gracilis* and bacterium were unclear. To identify the bacterium and its function, we performed isolation and sequencing experiments. The assembly of the complete genome met serious challenges: long and short tandem repeats and regions with high GC-content. Several sequencing technologies were used for the completion of the genome. Here we present the genome sequence of the isolate that is determined as *Paenibacillus* sp. The PCR product for 16S RNA isolated from the strain and *Euglena gracilis* culture was homogenous in sequence and was 100% identical to *Paenibacillus humicus* by BLAST.

No single tool gave the ideal assembly from Illumina reads in terms of both N50 and number of mis-assemblies (Table 1. Metrics of alternative draft assemblies), so the data of all the assemblies were used to verify one another. Assemblies based on Illumina reads including mate-pair reads could not resolve issues caused by long tandem copies of rRNA and extremely GC-rich regions (90–100%). Long Nanopore reads resolved those gaps and made it possible to complete the entire genome. Nanopore sequencing confirmed the correctness of scaffold assembly and clarified the sequences of tandem repeats; moreover, we found one plasmid. Issues and ambiguities are shown in Supplementary Table S1.

**Table 1:** Metrics of alternative draft assemblies.

Contig metrics	*SPAdes* v.3.6.0	*SPAdes* v.3.6.0	*Newbler* v.2.9 + HUMGGAT	*Velvet* v.1.2.0	*CLC* v.8.5	*CLC* v.8.5	*SPAdes* v.3.13.0	*Unicycler* v.0.4.8
Library type	Illumina paired-end	Illumina mate pair	Illumina paired-end; Illumina mate pair	Illumina paired-end	Illumina paired-end	Illumina mate pair	Illumina paired-end, Illumina mate pair, Oxford Nanopore	Illumina paired-end, Illumina mate pair, Oxford Nanopore
Largest contig	573,930	972,016	971,946	462,794	868,994	1883,967	1304,922	3347,597
Number of contigs ≥1000 bp	43	45	22	47	45	18	16	10
N50	259,753	240,878	427,030	179,795	317,660	1283,905	794,261	3347,597

The length of the genome *Paenibacillus* sp. RUD330 is 5.56 Mbp, and the average GC content is 59%. The mean coverage of the genome by the reads of three Illumina libraries was 467; 209 for Nanopore libraries. The length of the plasmid is 8.3 Kb, with the coverage by Nanopore reads at 429. We suppose that it is a two-copy plasmid.

The annotation of the genome was carried out with the *RAST* service (http://rast.nmpdr.org/), with *PGAP* 4.11*,* a Genbank tool, and *Prokka* 1.4.15 (https://github.com/tseemann/prokka) as an alternative (Table 2. Annotations characteristics). The deposited annotation (*PGAP*) revealed 4905 protein-coding genes, 8 copies of rRNA genes (5S, 16S, 23S), and 81 tRNA genes.

**Table 2:** Annotation characteristics.

Annotator	Genes (total)	CDS (total)	rRNA (5S, 16S, 23S)	tRNA	other ncRNA
RAST	5573	5468	8; 8; 8	81	0
PGAP 4.11	5014	4905	8; 8; 8	81	4
Prokka 1.4.15	5070	4919	8; 8; 8	83	44

## 2 Experimental design, materials, and methods

### 2.1 Species identity of *Euglena*

The species identity of *Euglena gracilis* has been confirmed using PCR and sequencing of mitochondrial *COI, COII*, chloroplast *PsaB* and *RbcL*.

### 2.2 Isolation and cultivation of strain

The medium with *Euglena* culture was streaked on Petri dishes with selective medium (macroelements, g/L: (NH_4_)_2_HPO_4_ - 1 g/L, KH_2_PO_4_ - 1 g/L, Na_2_C_6_H_5_O_7_ x 5H_2_O (citrate) - 0.8 g/L, MgSO_4_ - 0.2 g/L, CaCl_2_ - 0.02 g/L; microelement (mg/L): Fe_2_(SO_4_)_3_ x H_2_O - 3, MnCl_2_ x 4H_2_O - 1.8, CoCl_2_ x 6H_2_O - 1.3, ZnSO_4_ x 7H_2_O - 0.4, Na_3_Mo_4_ x 2H_2_O - 0.2, CuSO_4_ x 5H_2_O - 0.02; vitamins, ug/L: B1 - 20, B12 – 10; ethanol to 0.2 M; agar - 1.5%; antibiotics, ug/mL: ampicillin - 100, tetracycline – 25; pH 6.6–6.7). *Euglena* was eliminated, while the cohabiting bacterium showed resistance to antibiotics. Several colonies were picked and transferred to solid and liquid media. The bacteria were grown on liquid LB medium for NGS sequencing. The bacterial culture is available at M.V. Lomonosov Moscow State University, Department of Molecular Biology.

### 2.3 DNA isolation, libraries preparation, sequencing

DNA was isolated using the DIAtom DNAprep 100 kit (Izogen, Moscow).

The sequencing library with an insert size of 300–400 bp was prepared using the TruSeq DNA sample preparation kit (Illumina, USA) after the ultrasonic fragmentation of genomic DNA with Covaris S220. Two mate pair libraries with insert size ranges of 3000–4000 and 6000–8000 bp were created with the Nextera mate pair sample preparation kit (Illumina). The libraries were sequenced on Illumina HiSeq 2000, generating paired-end reads of 100 nt.

The library for Nanopore technology was prepared out of non-fragmented total genomic DNA using NEB Next Ultra II DNA library kit (NEB, UK) and Ligation Sequencing kit 1D (Oxford nanopore technologies, UK), barcoded using Native barcoding kit, and sequenced on MinION, R10 flowcell (Oxford nanopore technologies, UK).

### 2.4 Primary reads treatment

For Illumina reads *NextClip* v.0.8 [1] with default options was used to remove paired-end contamination in Nextera mate pair libraries. Adapters and regions of poor quality were trimmed using *Trimmomatic* v.0.32 [2] (PE-mode, -phred33, illuminaclip:Tru27.fa:2:30:10 leading:5 trailing:5 slidingwindow:4:12 minlen:40).

Nanopore reads with an average Phred quality score lower than 7 were discarded by *Guppy* 3.4.3 [3].

### 2.5 Genome assembly

*De novo* genome assembly with Illumina reads was performed with *Velvet* v.1.2.08 [4] (options: -exp_cov auto -cov_cutoff auto -ins_length 370 -min_contig_lgth 1000), *SPAdes* v.3.6.0 [5] (options: -m 200 –careful –hqmp), *CLC Genomics Workbench* v.8.5 (www.clcbio.com) (default settings), *Newbler* v.2.9 [6] where two assemblies were obtained: 1) only paired-end reads with options: -het -force -a 50 -ace -ar -cpu 15 -mi 95 -ml 20 -s 1000 -sc 1 -sio -sl 10 -ss 10; 2) paired-end and mate pair reads with options: -notrim -large -force -a 50 -ace -ar -cpu 15 -mi 95 -ml 20 -s 1000 -sc 1 -sio -sl 10 -ss 10, *HUMGGAT* (an in-house manual assembly finishing tool that helps to improve *Newbler* assemblies by working directly with the contig graph). Gaps between contigs that originated because of the repeats were filled by *GapFiller* 1.10 [7] with default options. *SPAdes* v3.13.0 with the "–careful" parameter was used to assemble the genome using Illumina paired end, Illumina mate pair, and Nanopore reads. *Unicycler* v.0.4.8 [8] with default parameters was used to assemble the genome from the same set of reads as *SPAdes* v3.13.0. Since *Unicycler* is incapable of utilizing mate pair reads, we provided them to *Unicycler* as unpaired single end reads. Manual manipulations with sequences and comparison of assemblies were carried out in *BioEdit* [9]. The detailed workflow was described in [10].

The circularity of the final assembly and absence of genomic regions that could be tandemly duplicated or lost due to mis-assemblies has been confirmed by mapping mate pair reads; moreover, we also checked for the absence of regions where the insert size of mate pair reads deviated from the average. To do this, the reads of the mate pair library with larger insert size 6–8 kB were mapped to the genome by *CLC Assembly Cell* 4.2 (www.clcbio.com), with the options set to map fully and without mismatches. The average insert sizes over all genome positions were visualized as a graph. The visual inspection indicated no regions with abrupt changes (more than 500 nt) in average insert sizes, which suggests that there were no mis-assemblies that resulted in large insertions or deletions.

## Declaration of Competing Interest

The authors declare that they have no known competing financial interests or personal relationships which have, or could be perceived to have, influenced the work reported in this article.

## Appendix Supplementary materials

Image, application 1

application 2

## References

[1] Leggett R.M., Clavijo B.J., Clissold L., Clark M.D., Caccamo M. (2014). NextClip: an analysis and read preparation tool for Nextera long mate pair libraries. Bioinformatics.

[2] Bolger A.M., Lohse M., Usadel B. (2014). Trimmomatic: a flexible trimmer for illumina sequence data. Bioinformatics.

[3] Oxford Nanopore Technologies, Guppy protocol, (2019). https://community.nanoporetech.com/protocols/Guppy-protocol/v/gpb_2003_v1_revq_14dec2018/linux-guppy.

[4] Zerbino D.R., Birney E. (2008). Velvet: algorithms for de novo short read assembly using de Bruijn graphs. Genome Res..

[5] Bankevich A., Nurk S., Antipov D., Gurevich A.A., Dvorkin M., Kulikov A.S., Lesin V.M., Nikolenko S.I., Pham S., Prjibelski A.D., Pyshkin A.V., Sirotkin A.V., Vyahhi N., Tesler G., Alekseyev M.A., Pevzner P.A. (2012). SPAdes: a new genome assembly algorithm and its applications to single-cell sequencing. J. Comput. Biol..

[6] Margulies M., Egholm M., Altman W.E., Attiya S., Bader J.S., Bemben L.a, Berka J., Braverman M.S., Chen Y.-.J., Chen Z., Dewell S.B., Du L., Fierro J.M., Gomes X.V., Godwin B.C., He W., Helgesen S., Ho C.H., Ho C.H., Irzyk G.P., Jando S.C., Alenquer M.L.I., Jarvie T.P., Jirage K.B., Kim J.-.B., Knight J.R., Lanza J.R., Leamon J.H., Lefkowitz S.M., Lei M., Li J., Lohman K.L., Lu H., Makhijani V.B., McDade K.E., McKenna M.P., Myers E.W., Nickerson E., Nobile J.R., Plant R., Puc B.P., Ronan M.T., Roth G.T., Sarkis G.J., Simons J.F., Simpson J.W., Srinivasan M., Tartaro K.R., Tomasz A., Vogt K.a, Volkmer G.a, Wang S.H., Wang Y., Weiner M.P., Yu P., Begley R.F., Rothberg J.M. (2005). Genome sequencing in microfabricated high-density picolitre reactors. Nature.

[7] Boetzer M., Pirovano W. (2012). Toward almost closed genomes with GapFiller. Genome Biol..

[8] Wick R.R., Judd L.M., Gorrie C.L., Holt K.E. (2017). Unicycler: resolving bacterial genome assemblies from short and long sequencing reads. PLoS Comput. Biol..

[9] Hall T.A. (1999). BioEdit: a user-friendly biological sequence alignment editor and analysis program for Windows 95/98/NT. Nucl. Acids. Symp. Ser..

[10] Shtratnikova V.Y., Schelkunov M.I., Donova M.V. (2017). Genome Sequencing of Steroid-Producing Bacteria with Illumina Technology. Methods Mol. Biol..

