# Supplementary material for: Complete genome assembly data of paenibacillus sp. RUD330, a hypothetical symbiont of euglena gracilis
Source: Data Brief. 2020 Jul 25;32:106070. doi: 10.1016/j.dib.2020.106070 (PMC7408339; doi:10.1016/j.dib.2020.106070)
Supplement: Supplementary file 1 [file mmc1.docx]

Supplementary Table S1. Used reads and assemblers. Ambiguities

| **Sequencer** | **Assembler** | **Reads type** | **Sign** |
| --- | --- | --- | --- |
| Illumina | Spades v.3.6.0 | Pair-end and mate pair with 3-4 Kb an 6-8 Kb inserts | MP+PE |
|  | Spades v.3.6.0 | Mate pairs with 3-4 Kb an 6-8 Kb inserts | MP |
|  | Spades v.3.6.0 | Mate pairs with 6-8 Kb inserts | MP6 |
|  | CLC v.8.5 | Pair-end and mate pair with 3-4 Kb an 6-8 Kb inserts | CLC |
|  | CLC v.8. | Mate pairs with 6-8 Kb inserts | CLC6 |
|  | Velvet v.1.2.0 | Pair-end | V |
|  | Newbler v.2.9 and HUMGGAT | Pair-end and mate pair with 3-4 Kb an 6-8 Kb inserts | H |
|  | Gapfiller | Assembly of Illumina reads |  |
| Nanopore | Unicycler 0.4.8 | Pair-end Illumina reads and Nanopore reads | U |
|  | Spades 3.13.0 | Pair-end and mate pair with 3-4 Kb an 6-8 Kb inserts Illumina reads and Nanopore reads | SP+N |

“+” denoted that this assembly version matching final complete variant

| **Position of start** | **Position of finish** | **First 20 nucleotides** | **Type of ambiguity** | **MP+PE** | **U** | **MP** | **MP6** | **CLC** | **CLC6** | **V** | **H** | **SP+N** |
| --- | --- | --- | --- | --- | --- | --- | --- | --- | --- | --- | --- | --- |
| 1885550 | 1885622 | GATCGATGTGTCTGCCAAGCT | Nucleotide substitutions |  | + |  |  |  |  |  |  |  |
| 2198480 | 2207580 | TCCTGTGGCGCCGGTAAGTCC | Complex ambiguity |  | + |  |  |  |  |  |  |  |
| 2446820 |  | GCGTGCAGTGCGCTTGAAGT | Repeats | + | + | + | + | + | + | + | + |  |
| 2467110 | 2467440 | CTGCCGCTGCCGCCGCCGAG | High GC content |  | + |  |  |  |  |  |  |  |
| 2894360 | 2894465 | GGTTTCGGCGCGCTCGCTGAC | Insertion and substitutions |  | + | + |  |  |  | + | + |  |
| 2998950 | 2999220 | CGAAGCCCGGCTCGCCGCCG | High GC content |  | + |  |  |  |  |  |  |  |
| 3785010 | 3785160 | GCTTCTTGCTGCTTCTTGCT | Inserts with different length |  | + |  |  |  |  |  |  |  |
| 3826470 | 3826540 | TTGCTTGTCTTGCTTGTCTT | Inserts with different length |  | + | + |  | + | + |  |  |  |
| 3971030 | 3971345 | GCCGGGAGCGGCTGCGCCGG | High GC content |  | + |  |  |  |  |  |  |  |
| 4093440 | 4093560 | CCAGCTTGCCGGCGGCAATG | Break in Unicycler assembly |  |  | + |  | + |  |  | + |  |
| 4145528 | 4145603 | CTGGCGCTCTGGACCTCAAG | Tandem repeats |  | + | + | + |  |  |  | + | + |
| 4146150 |  | ACCGCTACCTATCCGCATCT | Repeats | + | + | + | + | + | + | + |  | + |
| 4336364 | 4336393 | GGCTTCGCGCAGCCGCGCGG | Shift-frame indel and substitution, possible natural heterogenity |  | + |  |  |  |  |  |  |  |
| 4351390 | 4351730 | CAAGCAGAACGACCTCGCCG | Tandem repeats |  | + |  |  |  |  |  |  | + |
| 4483640 |  | GGTGCCGGGGTCGGCGTTGC | Tandem repeats |  |  | + |  | + | + | + | + |  |
| 4488690 | 4494256 | AAAAAAACCGCCAAGGGCGG | Ambiguity due to rRNA repeats |  | + |  |  |  |  |  |  | + |
| 4515362 | 4516067 | AAGCTGGCGCTCCGGCTTCC | Repeats. Nucleotide substitutions |  | + |  |  |  |  |  | + | + |
| 4512920 | 4517950 | CTACCATTGAACTACACCCG | Ambiguity due to rRNA repeats |  | + | + |  |  |  |  |  |  |
| 4687860 | 4688300 | CCCCCACCAAATCGCCACAA | Repeats | + | + |  |  | + |  |  |  |  |
| 4720560 | 4720900 | GCCTTAGGCCATGCTCCGCA | Tandem repeats |  | + |  |  |  |  |  |  |  |
| 4733700 | 4734220 | GGCCGCTCCAGGCCGCCGCC | High GC content |  | + |  |  |  |  |  |  |  |
| 4751840 | 4752460 | CTCCAAGGGGCTGCATGAGC | Tandem repeats |  | + |  |  |  |  |  |  |  |
| 4763980 | 4765430 | TCTGCTGACCCCTGGAAGCT | Tandem repeats | + | + | + | + | + | + | + |  |  |
| 4780320 | 4780700 | TGAAAGACATGCCTTTCGGC | Tandem repeats |  | + |  |  |  |  |  |  |  |
| 4805650 | 4806750 | ACTGTAGTCGCTCCCCGTAT | Tandem repeats |  | + | + | + |  |  |  |  |  |
| 4808140 | 4808300 | GGATGGCTGTCGCTCCCCGT | Tandem repeats |  | + | + | + |  |  |  |  |  |
| 4858050 | 4858180 | CGCGGAATCTTCCCGCCGAT | Tandem repeats |  | + |  |  |  |  |  |  |  |
| 4932070 | 4932170 | CCAGTCTCTCTCCTTTCTCT | Tandem repeats | + | + |  |  |  |  |  |  |  |
| 4986160 | 4986290 | CGAAAATCCACCCCGCCGCT | Tandem repeats |  | + |  |  |  |  |  | + |  |
| 4991810 | 4992720 | ACCTCTCCCTATCGTTATAT | Tandem repeats |  | + |  |  |  |  |  |  |  |
| 5190900 | 5191130 | CTCCAGCTCTCCAGCTCTCC | Tandem repeats |  | + |  |  |  |  |  |  |  |
| 5248840 | 5249350 | CAGTAGCTCCGGTGACTCCG | Tandem repeats | + | + | + | + | + |  | + |  |  |
| 5249620 | 5249960 | CTCCGGTTGCTCCTGTGACT | Tandem repeats |  | + | + | + | + |  | + |  |  |
| 5412540 | 5412810 | GTTCGCCAAAGGCGACGCGG | Tandem repeats |  | + | + |  |  |  |  |  |  |
| 11970 | 17260 | TAGCCTATGTGGCCGTGATA | Ambiguity due to rRNA repeats |  | + | + |  |  |  |  |  |  |
| 48830 | 49050 | GCCACCGGCGCCGCCGCGCT | High GC content |  | + |  |  |  |  |  |  |  |
| 49420 | 49820 | GAATATCGGACATAGGATCC | Tandem repeats |  | + |  |  |  |  |  |  |  |
| 110490 | 115930 | AAAGAAATGAAAAAAGTTAT | Ambiguity due to rRNA repeats |  | + | + |  |  |  |  |  |  |
| 172650 | 172990 | ACGGAAGCGGGGCAAATAAG | Tandem repeats |  | + | + | + |  |  |  |  |  |
| 251770 |  | CGATACATGAACATCTGGCC | Repeats | + | + | + | + | + | + | + | + |  |
| 263970 | 264280 | TCGCCGCGCAGCGAGACGCC | Insertion and substitutions |  | + |  |  |  |  |  |  |  |
| 270800 | 276220 | GGGGGCGTGATATATTATCT | Ambiguity due to rRNA repeats |  | + |  |  |  |  |  |  |  |
| 293590 | 294000 | CCGCTGCCGCTAAACTACGG | Tandem repeats |  | + |  |  |  |  |  |  |  |
| 319680 |  | CAACCGCAATCGCGGCGGCA | Insertion |  | + |  |  |  |  |  |  |  |
| 374140 |  | CTGAAAGGGCGAAGGCTTTG | Repeats | + | + | + | + | + | + | + |  |  |
| 391880 | 391980 | CTGCGGCAGCGCCAACGCCT | Tandem repeats |  | + |  |  |  |  | + |  |  |
| 441590 | 441810 | GGCCGGACCGGCAGGCCCGA | Tandem repeats |  | + | + | + |  |  |  |  |  |
| 496160 | 496400 | TCGCCGGCGCCGTGGGCGGG | Tandem repeats |  | + |  |  |  |  |  |  |  |
| 523970 | 524210 | AGTAGTGAGTCGATGAGAAA | Tandem repeats | + | + | + | + |  |  | + |  |  |
| 551770 | 551810 | GACGAAGCGGAAGCATGCGT | Tandem repeats | + | + | + | + | + | + | + |  |  |
| 566910 | 567040 | GTGGTGCGGGTAGATCGGGC | Tandem repeats |  | + |  |  |  |  |  |  |  |
| 692500 | 697840 | AATGATTAAGCTATAAATTC | Ambiguity due to rRNA repeats |  | + | + |  |  |  |  |  |  |
| 761190 | 761390 | GTGAGGGACGGTCAGGTATC | High GC content |  | + |  |  |  |  |  |  |  |
| 765960 | 774070 | GTTGCAATGAATGACTCGGC | Ambiguity due to rRNA repeats |  | + |  |  |  |  |  |  |  |
| 874310 | 874880 | GGCAGATTGGCTAGTCGCCA | Tandem repeats |  | + | + | + |  |  |  |  |  |
| 940990 | 941180 | ATACGGAATCGTCAAATAGC | Tandem repeats |  | + | + | + |  |  |  |  |  |
| 1053660 | 1053780 | GTGGAGTGGAGGCTCTCGCG | Tandem repeats |  | + | + | + | + | + | + | + |  |
| 1179390 | 1179890 | TGAACAAACCAAGCCATCCG | Tandem repeats |  | + |  |  |  |  |  |  |  |
| 1282050 | 1282390 | AGGAAACCCGGATGCTGAGG | Tandem repeats |  | + |  |  |  |  | + | + | + |
| 1326080 | 1326410 | GGCTTGTGAGCACGCAATAA | Tandem repeats |  | + |  |  | + | + | + |  | + |
| 1397310 | 1397440 | CCAAAAGCCAGAAAGCTTGA | Tandem repeats |  | + | + | + |  |  | + |  | + |
| 1405830 | 1406040 | AGCCTGATCATCGGTCGTAC | Tandem repeats |  | + | + | + |  |  |  |  | + |
| 1418840 | 1419170 | CCGGGAATAAGGGTTGAAAT | Tandem repeats | + | + | + | + | + | + | + |  | + |
| 1420210 | 1420520 | CGCTAAGAGCGGCAGCACCG | Tandem repeats |  | + |  |  |  |  |  |  |  |
| 1550240 | 1555660 | AAATAACACTTGCGCTAATC | Repeats |  | + |  |  |  |  |  |  |  |
| 1627180 | 1627340 | CATTTGGACAGGCGTTTTTT | Tandem repeats |  | + | + |  |  |  |  |  |  |
| 1703180 | 1703480 | CAAGCGGCATGATAGCGAGC | Tandem repeats | + | + |  |  |  | + |  |  |  |
| 1728620 | 1729700 | GTGGGAAGCAGGCAGCTGGC | Tandem repeats |  | + |  |  |  |  |  |  |  |
| 1790660 | 1790910 | GCTGATTTGAGGAATATCTG | Tandem repeats |  | + | + | + |  |  |  |  |  |
| 1791570 | 1791740 | GGCACCATCGCTGTCGTCGC | Tandem repeats | + | + | + |  | + |  | + |  |  |
